# Myelin oligodendrocyte glycoprotein antibody-associated disease: characterising clinical disease

**DOI:** 10.1007/s00415-018-8963-z

**Published:** 2018-07-10

**Authors:** M. D. Willis, N. P. Robertson

**Affiliations:** Institute of Psychological Medicine and Clinical Neuroscience, Cardiff University, University Hospital of Wales, Heath Park, Cardiff, CF14 4XN UK

Rather than being homogenous, it is becoming increasingly clear that central nervous system (CNS) neuroinflammatory diseases have a broad range of immunological aetiologies. In particular, the discovery of novel CNS antibodies has helped define new clinical disease entities. A greater understanding of the clinical phenomenology associated with these disorders will now be important to aid diagnosis and guide appropriate management.

Disease caused by one of these novel antibodies—directed towards myelin oligodendrocyte glycoprotein (MOG), has become a more intense focus of research as diagnostic assays have become more reliable and specific. MOG is expressed within the CNS on the surface of oligodendrocytes and myelin and associated with a range of adult and paediatric demyelinating disorders. Although acute disseminated encephalomyelitis (ADEM), optic neuritis (ON) and transverse myelitis (TM) are all phenotypes known to be related to MOG antibody (MOG-Ab) seropositivity, it is clear that further clinical characterisation and understanding of treatment outcomes is needed. It may be of particular relevance to distinguish these diseases from multiple sclerosis since standard disease modifying therapy may be ineffective and cause increased morbidity. Outcomes and treatment may also differ from aquaporin-4 antibody (AQP4) positive disease.

The three papers in this month’s journal club describe large cohorts of adults and children with MOG-Ab associated demyelinating disease. All aim to further characterise the clinical and radiological features of this disease alongside treatment outcomes. This represents an important step before establishing larger, systematic, prospective studies in addition to clinical trials of potential therapeutic regimens.

## Clinical spectrum and prognostic value of CNS MOG autoimmunity in adults: the MOGADOR study

This paper aimed to describe the clinical features and prognoses of patients with MOG-Ab associated disease after a first acute demyelinating event in a large French adult cohort. The clinical utility of MOG-Ab longitudinal analysis was also evaluated.

Patients aged ≥ 18 years presenting with at least one clinical demyelinating episode, and MOG-Ab seropositivity were retrospectively recruited. Demographic data were collected alongside clinical measures including Disability Status Scale (DSS), visual acuity (VA) and relapse history. In addition, medication use, biological aspects of disease and radiological findings were attained where available. Comparative data were obtained for an AQP4 positive cohort from a pre-existing database.

One hundred and ninety-seven patients were identified (M:F 1:1, 92.9% Caucasian) with a median age at presentation of 36.5 years. The most frequent phenotypes at disease onset were ON (60.9%) or myelitis (22.3%) either alone or in combination (7.6%). Other presentations included brainstem syndromes and encephalopathy with less frequent accompanying symptoms including neuropathic pain, fever, area postrema symptoms and seizures. Cerebrospinal fluid (CSF) pleocytosis was observed in 44.2% of patients, most frequently present in those presenting with myelitis. CSF oligoclonal bands (OCBs) were only seen in a minority (5.7%). After a median follow-up of 15.8 months, 42.1% patients relapsed. The cumulative risk to reach a relapse after 2 and 5 years was 44.8 and 61.8%, respectively, for patients with ON or myelitis at onset. At last follow-up, 24.7 and 10.4% patients presenting with non-ON phenotypes reached a DSS score of 3.0 and 6.0, respectively. Of patients presenting with ON, only 8.1% reached VA of 20/100 at the last follow-up. In comparison to the AQP4 cohort, MOG-Ab patients had a lower risk to reach a first relapse and of attaining a DSS score of 3 or visual acuity 20/100.

MOG-Ab patients had distinctive pontine and thalamic lesions on MRI imaging with cortical involvement and leptomeningeal enhancement also seen. Myelitis lesions (either alone or with ON) at onset were longitudinally extensive in 84.4 and 55.6%, respectively. MOG-Ab titres were higher in relapse compared with remission and in those with monophasic disease only 2 (18.2%) became seronegative.

*Comment* This study adds to knowledge regarding the spectrum of clinical phenotypes in adult patients with MOG-Ab positive disease. In particular, MOG-Ab patients have a milder disease course when compared with AQP4 positive patients. Interestingly, specific findings on MRI were observed in patients with MOG-associated disease, which may help identify these patients at an earlier stage. The authors acknowledge that as not all patients were diagnosed at onset of disease, differing treatment and managements may have potentially affected the disease course. In addition, the timing of MRI and serum samples were not standardised.

*Cobo-Calvo A et al. Neurology. 2018;90(21):e1858–e1869*

## Clinical course, therapeutic responses and outcomes in relapsing MOG antibody-associated demyelination

This paper aimed to characterise the clinical course, treatment and outcomes in both adult and paediatric MOG-Ab positive patients with relapsing disease. Fifty-nine (40 female, 73% Caucasian) patients with relapsing demyelination were identified from 25 Australasian centres (adult > 16 years, *n* = 26; paediatric ≤ 16, *n* = 33). Data were retrospectively collected for annualised relapse rates (ARR) pre- and post-treatment, therapeutic responses and Expanded Disability Status Scale (EDSS) outcomes.

The median age of onset for the total cohort was 12 years (adults 37 years, paediatrics 6) with an infectious prodrome present in 47%. Mean EDSS at initial presentation was 5.1 (adults 4.4, paediatric 5.6). In total, 218 demyelinating episodes were identified during an average follow-up of 61 months. Two demyelinating episodes were observed in 33.9% patients and 66.1% patients had ≥ 3.

ADEM was the most common initial presentation in children (36%) followed by bilateral ON (BON, 24%) and unilateral ON (UON, 15%). Adults presented primarily (73%) with ON (BON 42%, UON 31%). TM was said to be less common. Similarly, across all relapsing episodes, ON was most common; UON (35%), BON (19%). ADEM was common in children (15%) but rare in adults (2%). TM was less common in the whole cohort (11%) but more prevalent in adults. Relapsing ON was the main relapsing clinical syndrome in the total cohort (29%) followed by neuromyelitis spectrum disorder (NMOSD, 25%).

A CSF mononuclear pleocytosis and elevated protein was observed in 58 and 37% patients respectively. OCBs were positive in 11%. No brain lesions were observed in 48% of MRI scans. With respect to treatment, all immunosuppressant treatments used were associated with a reduction in ARR with maintenance prednisone having the lowest treatment failure rate. Residual disability was observed in 58%, which was more likely in those with myelitis. The mean EDSS at latest follow-up for the total cohort was 1.3.

*Comment* This study increases knowledge about the clinical phenotype and outcomes in both adults and children with MOG-Ab associated disease. The positive response to immunosuppressive treatment is encouraging as is the finding that the majority of patients have a positive outcome. Limitations of the study include its retrospective design and the limited numbers of patients in some treatment groups. In addition, some patients were on several therapeutic agents simultaneously, which should suggest caution when interpreting responses to individual treatments. The authors conclude that MOG antibodies should be considered in all childhood-onset demyelination and in adults with an atypical phenotype for MS, particularly if isolated or recurrent ON.

*Ramanathan et al. J Neurol Neurosurg Psychiatry. 2018;89(2):127–137*

## Disease course and treatment responses in children with relapsing myelin oligodendrocyte glycoprotein antibody-associated disease

This study focused on a MOG-Ab positive paediatric population with relapsing disease. The aims of the study were to describe the first attack features, paraclinical characteristics, disease course and responses to different treatment strategies.

In this retrospective European multicentre study, demographic, clinical and radiologic data were collected from patients < 18 years old with a relapsing demyelinating syndrome and MOG-Ab seropositivity. One hundred and two patients were identified with a median age of 7 years (M:F 1.0:1.8) with the majority of patients being of Caucasian ethnicity. A total of 464 demyelinating events were reported in the cohort over a median length of follow-up of 5 years.

The most frequent demyelinating phenotype at onset was ADEM (52%) followed by ON (40.2%; BON 43.9%, UON 36.6%). Simultaneous ON and TM was observed in 19.5% of patients. Patients 9 years or younger were more likely to have clinical and radiological changes affecting the brain, whereas events in patients older than 9 years were more likely to affect the optic nerve and/or TM. A CSF lymphocytosis was observed in 58.7 and 11.1% had positive OCBs. Erythrocyte sedimentation rate was increased in 58.3% patients.

Immunomodulatory or immunosuppressive treatment was administered to 51% of patients. Patients received either one treatment (53.8%), two treatments (30.7%) or three or more treatments (13.5%). Although patients relapsed during all treatments, regular IVIg, rituximab, mycophenolate mofetil and azathioprine were all associated with a reduction in ARR. Reduction in the EDSS score was also observed in those patients receiving IVIg. Patients receiving immunotherapy had more relapses and worse EDSS than untreated patients (median 1.5 vs. 1.0 and 3.0 vs. 1.0) respectively. A good recovery, defined as an ARR and EDSS of 0 at last follow-up was reported in 31.3% patients.

*Comment* The phenotype and treatment outcomes in paediatric patients with MOG-Ab associated disease were further characterised in this paper. Interestingly, an age-dependent phenotype was observed, which the authors suggest may be related to the normal physiologic processes of white matter maturation. Although treated patients in this study had a worse outcome, this likely reflects a treatment paradox whereby patients with more severe initial disease are likely to be considered candidates for intervention. Although the number of patients in each treatment group was relatively small, the finding that IVIg was superior to other treatments and that injectable MS drugs were not associated with improvement is clinically important.

*Hacohen et al. JAMA Neurol. 2018;75(4):478–487*

