# Correction to: Myelin oligodendrocyte glycoprotein antibody-associated disease: characterising clinical disease

**DOI:** 10.1007/s00415-018-9019-0

**Published:** 2018-08-27

**Authors:** M. D. Willis, N. P. Robertson

**Affiliations:** Institute of Psychological Medicine and Clinical Neuroscience, Cardiff University, University Hospital of Wales, Heath Park, Cardiff, CF14 4XN UK

## Correction to: Journal of Neurology (2018) 265:1950–1952 10.1007/s00415-018-8963-z

The original version of this article unfortunately contained a mistake. The copyright line was incorrect in the HTML version. The correct copyright line should be “The Author(s) 2018”.

